# Association between the systemic inflammation response index and the prognosis of patients with myocardial infarction: a systematic review and meta-analysis

**DOI:** 10.3389/fcvm.2026.1755442

**Published:** 2026-02-16

**Authors:** Yedan Wu, Rong Zheng, Yuling Lin, Zhiqing Shen, Hong Shi

**Affiliations:** 1 School of Basic Medical Sciences, Zhejiang Chinese Medical University, Hangzhou, China; 2 The People’s Hospital of Fujian Traditional Medical University, Fuzhou, Fujian, China; 3 Fujian University of Traditional Chinese Medicine, Fuzhou, Fujian, China

**Keywords:** immunology, meta-analysis, myocardial infarction, prognosis, systemic inflammation response index

## Abstract

**Background:**

The systemic inflammation response index (SIRI), a novel biomarker integrating neutrophil, monocyte, and lymphocyte counts, has been implicated in cardiovascular disease prognosis. This study aimed to systematically evaluate the association between SIRI and clinical outcomes in patients with myocardial infarction (MI).

**Methods:**

A comprehensive literature search was conducted across multiple databases up to July 2025. Observational studies reporting odds ratios (ORs) with 95% confidence intervals (CIs) for the association between SIRI and post-MI outcomes were included. Pooled ORs were calculated using random-effects models. Heterogeneity and publication bias were assessed.

**Results:**

Twelve comparative groups (6,751 participants) showed that elevated SIRI was possibly associated with an increased risk of major adverse cardiovascular events (MACE) (OR = 1.42, 95% CI: 1.27–1.58). SIRI was also potentially associated with higher all-cause mortality (OR = 1.28), stroke (OR = 1.11), subsequent AMI (OR = 1.21), and the Gensini score (OR = 6.89). Significant heterogeneity was observed for some outcomes. Subgroup analyses indicated that study sample size and SIRI cut-off values were potential sources of heterogeneity.

**Conclusion:**

An elevated SIRI is consistently associated with an increased risk of adverse clinical outcomes in patients with MI, underscoring its potential value as a readily accessible prognostic biomarker for risk stratification.

**Systematic Review Registration:**

https://www.crd.york.ac.uk/PROSPERO/, identifier CRD420251169048.

## 1 Introduction

Acute myocardial infarction (AMI) remains a leading cause of global cardiovascular mortality and morbidity, posing a major public health challenge (1). Significant advances in reperfusion therapies, such as percutaneous coronary intervention and thrombolysis, alongside improved pharmacological management, have markedly reduced acute-phase mortality in recent years (2). However, the risk of subsequent major adverse cardiovascular events (MACE), including post-infarction heart failure, remains unacceptably high (3). Although dual antiplatelet therapy (DAPT) with varying treatment durations is currently widely recommended in clinical practice due to its lower risk of adverse events, this regimen still carries the drawback of increasing bleeding risks, which can severely impact patients' long-term prognosis and quality of life (4). Consequently, elucidating the underlying mechanisms of AMI onset and progression, and identifying novel targets for effective risk prediction and intervention, continue to be central focuses in cardiovascular research.

Inflammation contributes significantly to coronary artery disease (CAD) (5). In recent years, growing interest has been directed toward understanding the critical role of inflammation in the development and progression of cardiovascular diseases (CVDs), including atherosclerosis, myocardial infarction, heart failure, and pericarditis (6). Multiple studies have found that reducing inflammation levels may have beneficial effects in patients at high risk of CVDs by using anti-inflammatory drugs, supporting intervention through inflammatory pathways as an effective strategy for secondary prevention of CVDs (7). Excessive or dysregulated inflammation can exacerbate myocardial injury and promote maladaptive ventricular remodeling, thereby driving disease progression (8). Against this backdrop, biomarkers capable of precisely quantifying the systemic inflammatory status, such as the Systemic Inflammation Response Index (SIRI), have attracted growing interest. As a novel composite inflammatory index, SIRI integrates neutrophil, monocyte, and lymphocyte counts, offering a more comprehensive reflection of the body's inflammatory-immune balance (9). Recent studies (10, 11) have revealed significant associations between SIRI and both the severity of coronary artery disease and the risk of major adverse cardiovascular events following myocardial infarction, highlighting its promising potential as a risk prediction tool.

However, the prognostic value of SIRI in specific myocardial infarction populations, as well as its advantages over traditional inflammatory markers, require further clarification through additional evidence. Therefore, this study aims to thoroughly investigate the association between SIRI and long-term clinical outcomes in patients with myocardial infarction. We anticipate that this work will contribute a novel and effective biomarker for risk stratification and prognosis assessment in myocardial infarction, while also providing a theoretical foundation for future precision treatment strategies targeting inflammatory pathways.

## 2 Materials and methods

### 2.1 Literature search

This study was conducted and reported in accordance with the Preferred Reporting Items for Systematic Reviews and Meta-Analyses (PRISMA 2020) guidelines (12). The research protocol was prospectively registered in the International Prospective Register of Systematic Reviews (PROSPERO: CRD420251169048). Two reviewers (WYD and ZR) collaboratively designed the search strategy. Each reviewer independently selected subject headings and free-text terms and performed literature searches across major databases—PubMed, Embase, Web of Science, and the Cochrane Library—covering all records available up to July 3, 2025. The search strategy incorporated a comprehensive range of keywords, including: “Myocardial Infarctions”, “Heart Attack”, “Heart Attacks”, “Myocardial Infarct”, “Myocardial Infarcts”, “Cardiovascular Stroke”, “Cardiovascular Strokes”, “Systemic Inflammation Response Index”, and “SIRI.” Supplementary Table S1 presents the literature search strategy.

### 2.2 Study selection

Eligible studies met the following requirements: (1) MI was confirmed by cardiac biomarkers or imaging evidence; (2) the prognostic significance of the systemic inflammation response index (SIRI) was evaluated; (3) sufficient information was provided to obtain odds ratios (ORs) with 95% confidence intervals (CIs); (4) participants were grouped into high- and low-SIRI categories based on defined thresholds; and (5) the study was available in full-text form. Exclusion criteria were: (1) non-original publications (reviews, editorials, conference abstracts, case reports, and letters); (2) incomplete data for OR or CI estimation; (3) absence of prognostic outcomes; and (4) duplicated or overlapping data.

The screening process was conducted independently by two investigators (WYD and ZR), who examined the titles and abstracts of all retrieved records, retrieved full texts, and determined study eligibility. Any disagreements were resolved by consensus during the study selection process.

### 2.3 Data extraction

Two investigators (WYD and ZR) independently extracted the data, and any inconsistencies were resolved by consensus among all authors. Extracted data included study characteristics such as first author, study period, location, design, population, treatment type, sample size, sex distribution, mean or median age, SIRI threshold, outcomes, and ORs with 95% CIs for MI.

### 2.4 Quality assessment

The Newcastle-Ottawa Quality Assessment Scale (NOS) (13) was employed to evaluate study quality across three aspects: selection, comparability, and outcome. A maximum of 9 points could be assigned, and studies scoring 7 or above were deemed high quality.

### 2.5 Statistical analysis

The combined OR and corresponding 95% CI were calculated to assess the impact of SIRI on the prognosis of MI patients. SIRI = (monocyte count × neutrophil count)/lymphocyte count was used to calculate the value of SIRI in included studies (14). Cochran's *Q* test and Higgins' *I*^2^ statistic were used to quantify heterogeneity. A threshold of *I*^2^ > 50% or *p* < 0.1 was defined as indicating significant variability across studies. All data were pooled using a random-effects model. In addition, to verify the stability of the pooled estimates and to explore possible contributors to heterogeneity, sensitivity and subgroup analyses were carried out. The presence of publication bias was evaluated through visual inspection of funnel plots and statistically tested using Egger's test. A *p*-value <0.05 was considered statistically significant. Data analyses were performed using Stata 18.0 and RevMan 5.4 software packages.

## 3 Results

### 3.1 Study characteristics

**Figure 1:**
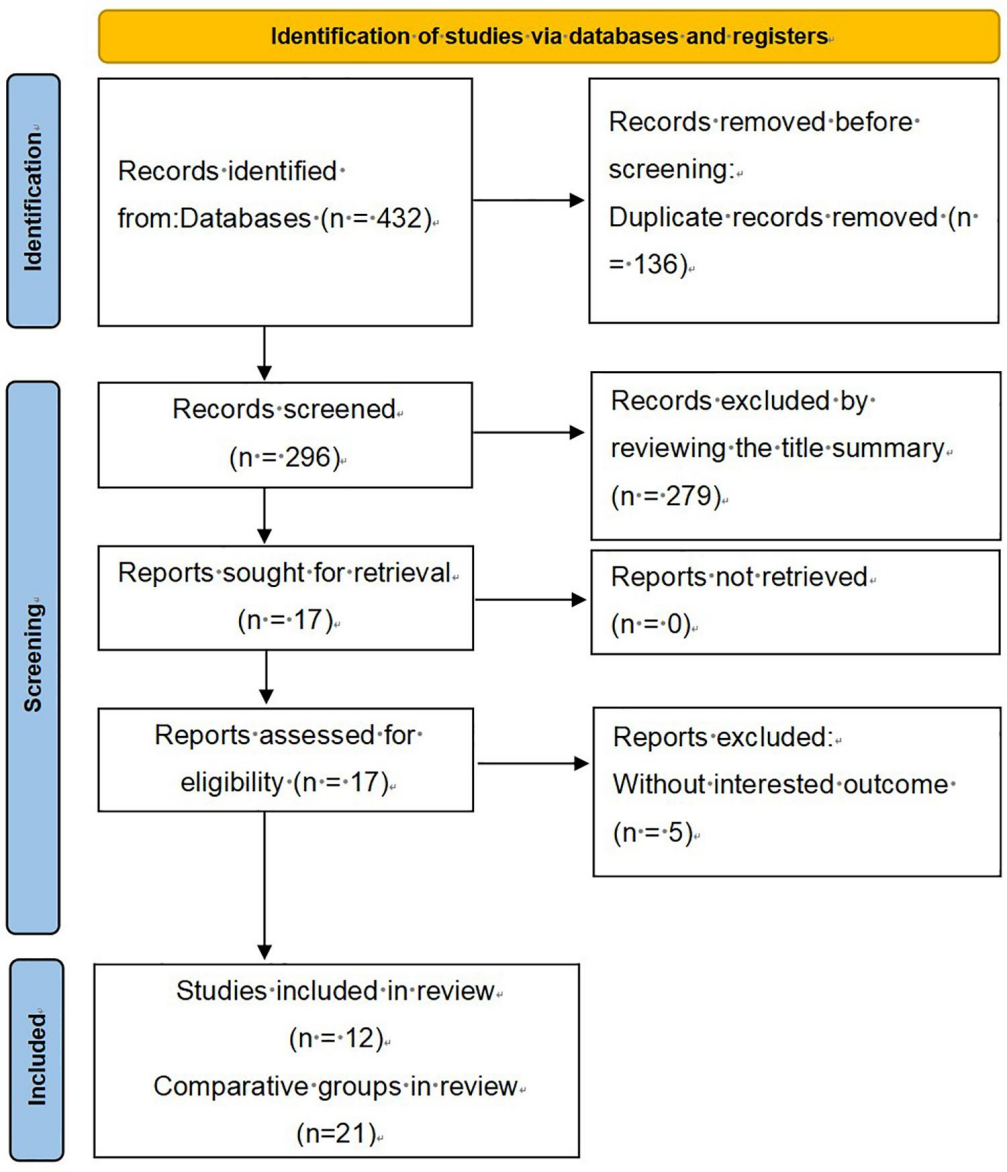
PRISMA literature screening procedure.

The database search initially yielded 432 records. After excluding 136 duplicate entries, 296 studies were retained for screening. Based on title and abstract review, 279 studies were excluded. Subsequently, 12 full-text articles were assessed for eligibility, and 5 were excluded because they did not provide adequate information for survival analysis. As there were several experimental studies on SIRI with different cut-off values in the included articles, they were extracted separately, and 21 comparison groups were finally obtained, encompassing a total of 96,559 patients (14–25) (Figure 1).

Among the eligible 21 comparative groups, 1 group was conducted in India and 2 in the USA, while the remaining 18 were conducted in China. Among them, 3 cohort groups were retrospective cohorts, whereas the remaining 18 were prospective. The cohort studies included in this analysis were all published in English-language journals between 2021 and 2025. The SIRI index was applied in all studies, with participants divided into high- and low-SIRI groups for the purpose of analysis. Regarding treatment, 12 groups reported Percutaneous Coronary Intervention (PCI), and 9 groups did not report treatment information. Following the extraction of prognostic outcome data, the analysis included multiple study groups investigating the association between SIRI and various post-MI outcomes. Specifically, 12 groups assessed Major Adverse Cardiovascular Events (MACE), 6 groups assessed all-cause mortality, 3 groups assessed stroke, 3 groups assessed the Gensini score, and 2 groups assessed subsequent AMI. Among these, three groups concurrently evaluated both mortality and stroke, while two groups evaluated both MACE and AMI. The main features of the included studies are presented in Table 1.

**Table 1:** The characteristics of studies.

Comparative groups	study period	Country	Study design	Participant source	Treatment method	No. of patients	Female (%)	Age (Mean ± SD)	SIRI cut-off	Outcome
Jin et al. (14)	2006–2017	China	Prospective cohort	CVDs in Kailuan general cohort	N/R	85,154	/	51.3	0.13	Mortality, Stroke
Jin et al. (14)	2006–2017	China	Prospective cohort	CVDs in Kailuan general cohort	N/R	85,154	/	51.3	0.60	Mortality, Stroke
Jin et al. (14)	2006–2017	China	Prospective cohort	CVDs in Kailuan general cohort	N/R	85,154	/	51.3	1.07	Mortality, Stroke
Han et al. (15)	2016–2017	China	Prospective cohort	ACS patients undergoing PCI	PCI	1,724	23.26	60	0.63	MACE
Han et al. (15)	2016–2017	China	Prospective cohort	ACS patients undergoing PCI	PCI	1,724	23.26	60	1.02	MACE
Li et al. (16)	2019–2021	China	Prospective cohort	Initially diagnosed CAD patients	PCI	959	48.18	61.35	2.47	MACE
Liu et al. (17)	2016–2020	China	Prospective cohort	STEMI patients undergoing primary PCI	PCI	216	18.82	64	4.15	MACE
Qu et al. (18)	2019–2021	China	Prospective cohort	STEMI patients undergoing PCI	PCI	1,312	19.3	58.4	1.58	MACE
Qu et al. (18)	2019–2021	China	Prospective cohort	STEMI patients undergoing PCI	PCI	1,312	19.3	58.4	3.28	MACE
Qu et al. (18)	2019–2021	China	Prospective cohort	STEMI patients undergoing PCI	PCI	1,312	19.3	58.4	7.8	MACE
Wang and Chen (19)	2012–2019	USA	Prospective cohort	Critically ill AMI patients in ICU	N/R	4,291	37.3	69.4	2.9	Mortality
Wang and Chen (19)	2012–2019	USA	Prospective cohort	Critically ill AMI patients in ICU	N/R	4,291	37.3	69.4	4.6	Mortality
Wei et al. (20)	2018–2022	China	Prospective cohort	AMI patients undergoing PCI	PCI	310	20	62.8	1.72	MACE
Wei et al. (20)	2018–2022	China	Prospective cohort	AMI patients undergoing PCI	PCI	310	20	62.8	3.68	MACE
Guo et al. (21)	2020–2023	China	Cross-sectional	STEMI patients	N/R	1,258	27.7	62.22	0.87	Gensini Score
Guo et al. (21)	2020–2023	China	Cross-sectional	STEMI patients	N/R	1,258	27.7	62.22	1.60	Gensini Score
Guo et al. (21)	2020–2023	China	Cross-sectional	STEMI patients	N/R	1,258	27.7	62.22	2.86	Gensini Score
Hou et al. (22)	2015–2022	China	Prospective cohort	MINOCA patients	N/R	259	36.7	58.41	1.743	MACE
Ma and Geng (23)	2018–2019	China	Prospective cohort	Elderly (>60 yrs) ACS patients after PCI	PCI	960	32.4	68	N/R	MACE, AMI
Marchi et al. (24)	N/R	Italy	Prospective cohort	STEMI patients	PCI	105	30	70	4.9	Mortality
He et al. (25)	2015–2020	China	Prospective cohort	ACS patients with obstructive sleep apnea	PCI	1,011	12.7	56.5	1.16	MACE, AMI

### 3.2 Study quality

Each of the eight studies received a NOS score ranging from seven to eight, indicating that they were of high quality (Supplementary Table S2).

### 3.3 Meta-analysis results

#### 3.3.1 SIRI and MACE

**Figure 2:**
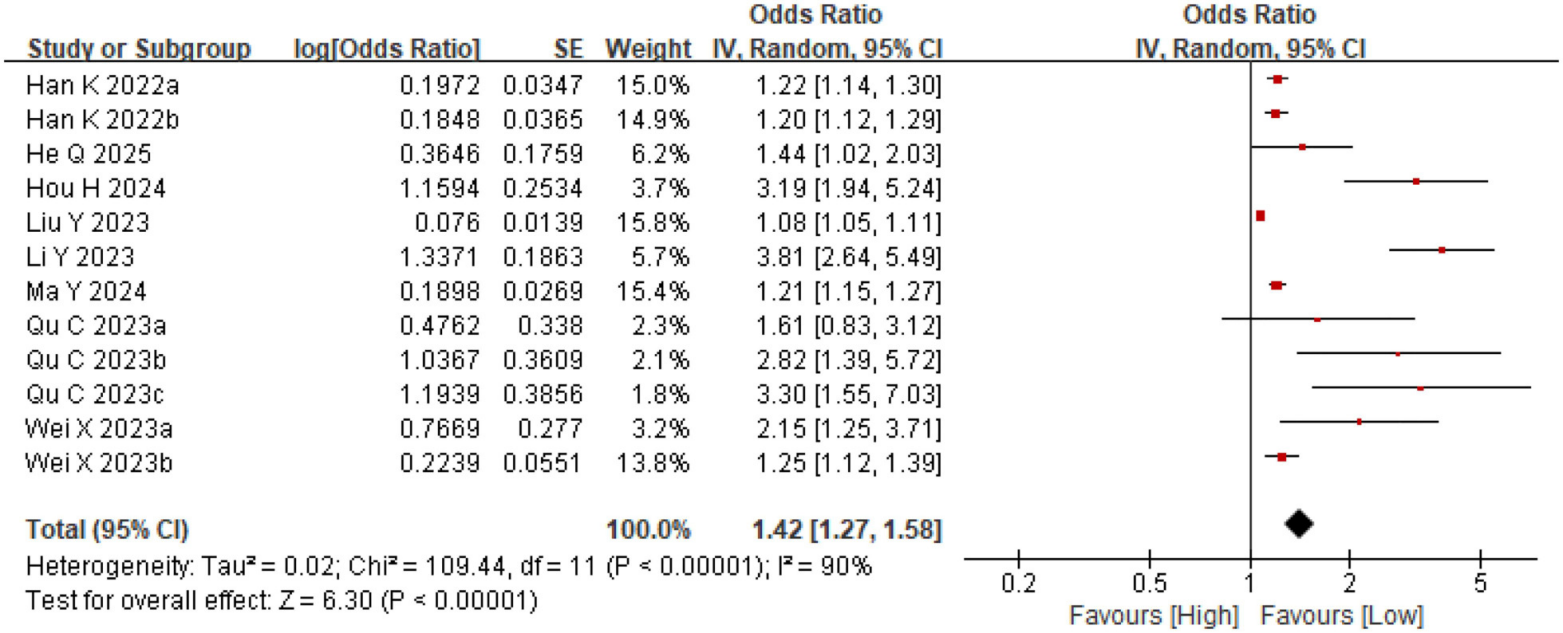
Forest plots for the association between SIRI and MACE.

We examined the association between the Systemic Inflammation Response Index (SIRI) and major adverse cardiovascular events (MACE) across twelve comparative groups involving 6,751 participants. Significant heterogeneity (*I*^2^ = 90%, *p* < 0.0001) warranted the use of a random-effects model in the analysis (Figure 2). The analysis demonstrated that elevated SIRI levels were potentially associated with an increased risk of MACE (OR = 1.42, 95% CI: 1.27–1.58; *p* < 0.0001) (Figure 2). Subgroup analyses of MACE were conducted based on mean age, sample size, and SIRI cut-off values, and SIRI had predictive value for MACE after MI in all subgroups, as detailed in Supplementary Table S3.

#### 3.3.2 SIRI and other prognostic outcome measures

**Figure 3:**
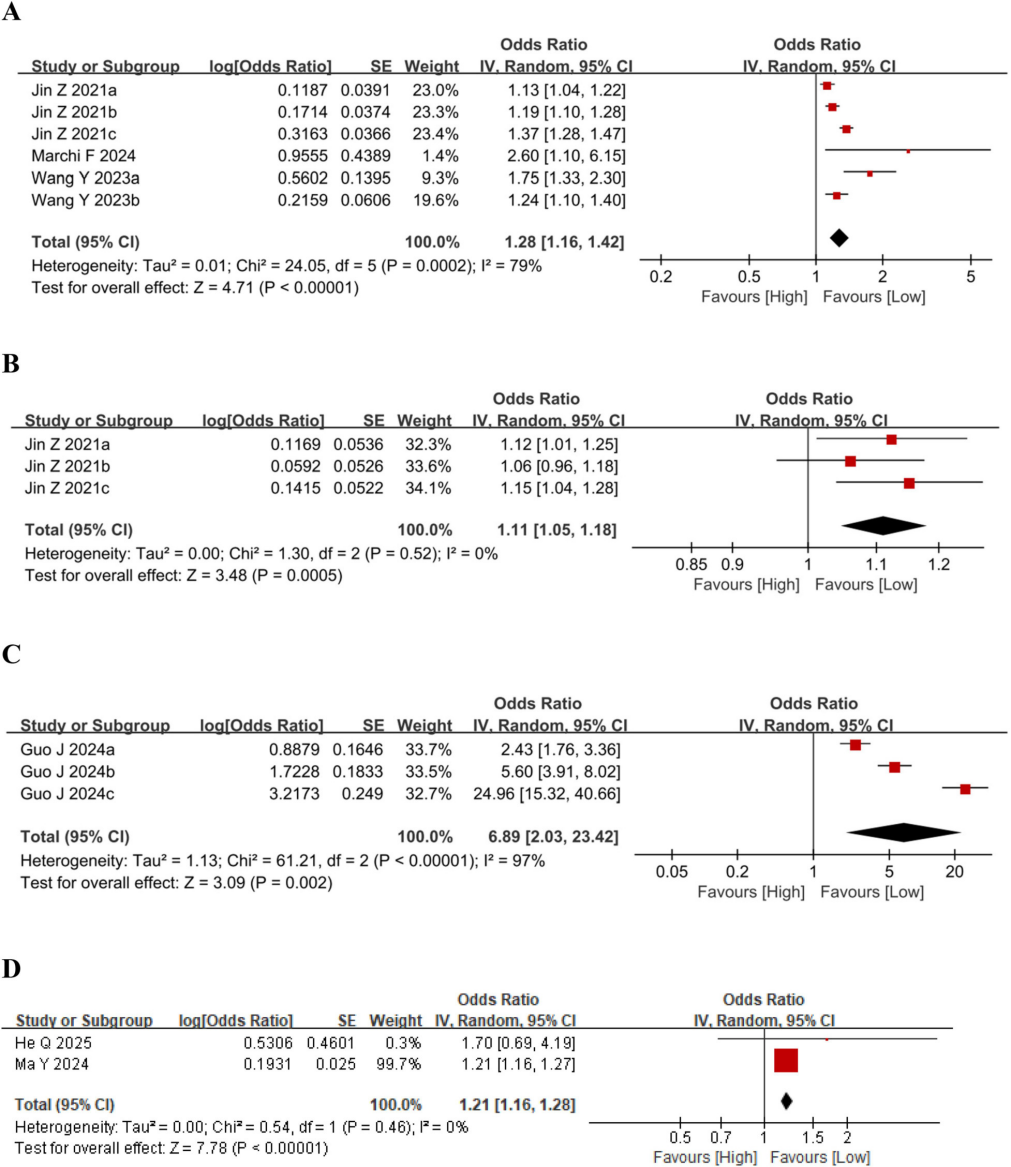
**(A)** Forest plots for the association between SIRI and mortality; **(B)** forest plots for the association between SIRI and stroke; **(C)** forest plots for the association between SIRI and gensini score; **(D)** forest plots for the association between SIRI and AMI.

We evaluated the association between the Systemic Inflammation Response Index (SIRI) and mortality across six comparative groups involving 89,550 participants. Substantial heterogeneity was observed among the studies (*I*^2^ = 79%, *p* < 0.0001); thus, a random-effects model was employed (Figure 3A). The results indicated that higher SIRI levels were moderately associated with increased mortality (OR = 1.28, 95% CI: 1.16–1.42, *p* < 0.0001).

Additionally, three studies examined the relationship between SIRI and stroke, revealing that elevated SIRI was associated with a higher risk of stroke (OR = 1.11, 95% CI: 1.05–1.18, *I*^2^ = 0%, *p* < 0.0005) (Figure 3B). Another three studies assessed the correlation between SIRI and the higher Gensini score, demonstrating that higher SIRI values were linked to moderately elevated Gensini scores (OR = 6.89, 95% CI: 2.03–23.42, *I*^2^ = 97%, *p* = 0.002) (Figure 3C). Finally, two studies explored the association between SIRI and acute myocardial infarction (AMI), showing that increased SIRI was related to a higher AMI risk (OR = 1.21, 95% CI: 1.16–1.28, *I*^2^ = 0%, *p* < 0.0001) (Figure 3D).

### 3.4 Sensitivity analysis

**Figure 4:**
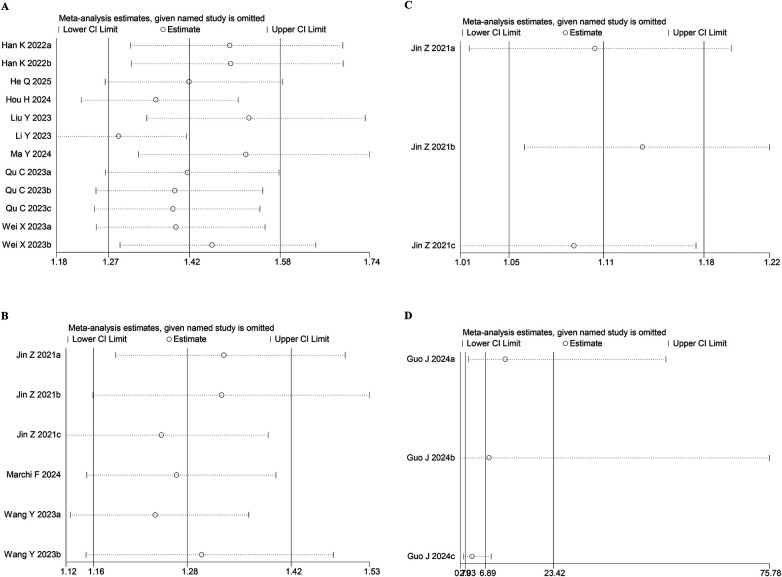
Sensitivity analysis of **(A)** MACE, **(B)** mortality, **(C)** stroke, **(D)** gensini score.

To evaluate the robustness of the association between baseline SIRI and clinical outcomes, a leave-one-out sensitivity analysis was conducted. The results showed consistent effect sizes within the original confidence intervals after the sequential removal of individual studies, indicating that none disproportionately affected the summary estimates for MACE (Figure 4A), mortality (Figure 4B), stroke (Figure 4C), or Gensini score (Figure 4D). This supports the overall reliability of the meta-analysis.

### 3.5 Publication bias

**Figure 5:**
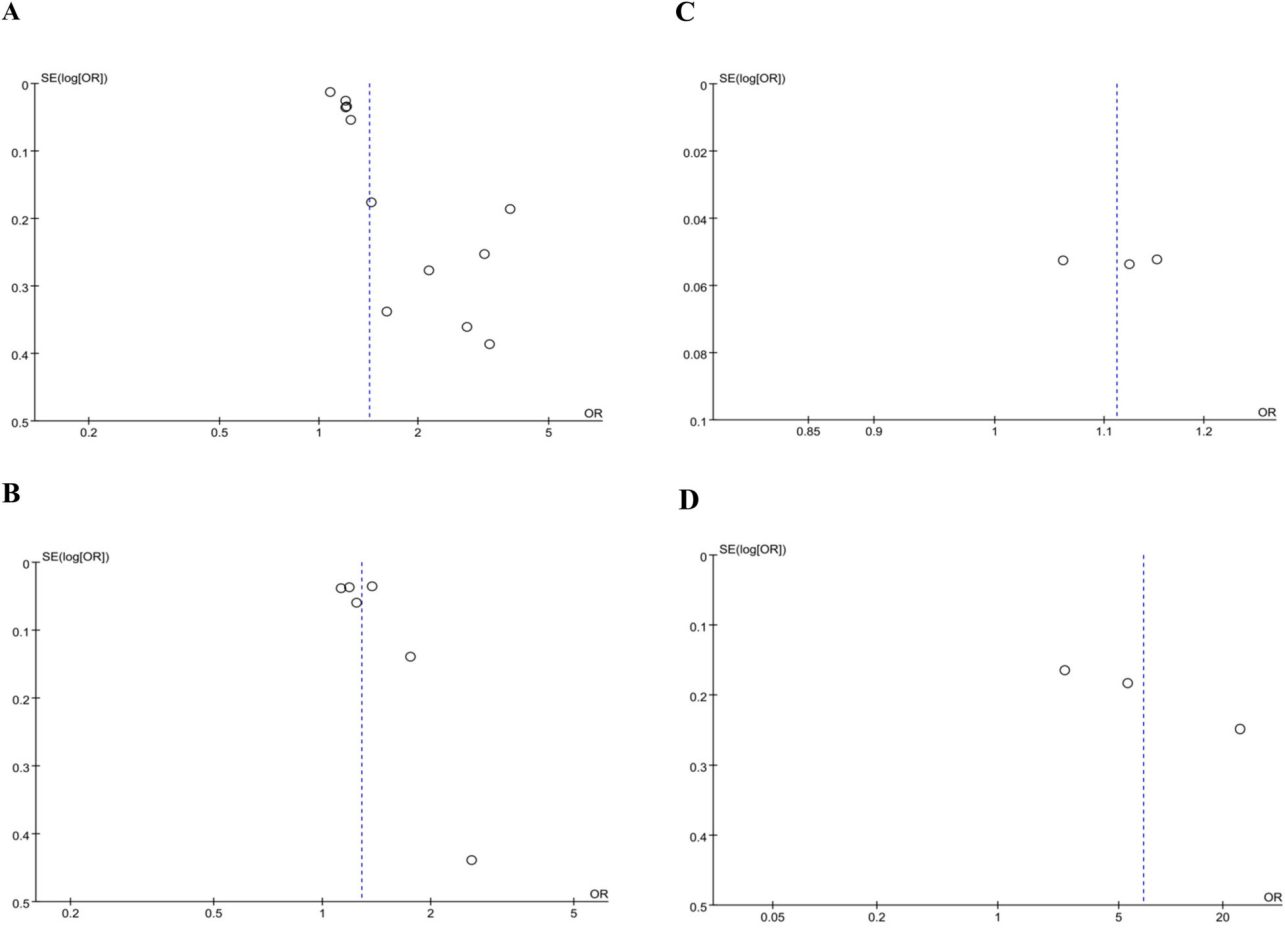
Funnel plot for the evaluation of publication bias for **(A)** MACE, **(B)** mortality, **(C)** stroke and **(D)** gensini score.

We examined potential publication bias through visual inspection of funnel plots and by performing Egger's regression test. Analysis for MACE revealed significant bias, as suggested by an asymmetric funnel plot and confirmed by Egger's test (*p* = 0.0001; Figure 5A). However, no significant publication bias was identified for mortality (*p* = 0.269; Figure 5B), stroke (*p* = 0.959; Figure 5C), or the Gensini score (*p* = 0.113; Figure 5D). Analyses for other outcomes were not feasible due to the limited number of included studies (<3).

## 4 Discussion

Cardiovascular diseases (CVDs) continue to be among the leading causes of mortality globally and are associated with substantial health burden and increased healthcare costs (26). Patients with established atherosclerotic cardiovascular disease (ASCVD) face a heightened risk of recurrent cardiovascular events, including myocardial infarction, stroke, and cardiovascular death. Over the past several decades, inflammation has emerged as a key contributor to this residual risk (27–29).

This meta-analysis indicates that an elevated Systemic Inflammation Response Index (SIRI) is moderately associated with an increased risk of major adverse cardiovascular events (MACE) (OR = 1.42), all-cause mortality (OR = 1.28), stroke (OR = 1.11), subsequent acute myocardial infarction (OR = 1.21), and higher coronary severity as measured by the Gensini score (OR = 6.89) in patients with myocardial infarction. Despite significant heterogeneity in some analyses, the findings were robust in sensitivity analyses. These results underscore the strong prognostic value of SIRI, a readily available biomarker derived from routine blood counts, for risk stratification in MI patients. The findings suggest that SIRI can help identify high-risk individuals who may benefit from more intensive monitoring and tailored management strategies, potentially including anti-inflammatory therapies, to improve long-term cardiovascular outcomes.

Based on the subgroup analysis data, the sources of heterogeneity in the association between SIRI and MACE can be partially elucidated. Significant heterogeneity was observed across studies, particularly when stratified by sample size and SIRI cut-off values. Studies with smaller sample sizes (<960) showed a higher pooled OR (1.86) and greater heterogeneity (*I*^2^ = 95%), compared to larger studies (≥960) with a lower OR (1.25) and moderate heterogeneity (*I*^2^ = 57%). Similarly, a higher SIRI cut-off (>2) was associated with a stronger effect estimate (OR = 1.85) and high heterogeneity (*I*^2^ = 94%), whereas a lower cut-off (≤2) yielded a more moderate OR (1.40) with lower heterogeneity (*I*^2^ = 75%). These variations suggest that differences in study size and SIRI threshold definitions are key contributors to heterogeneity, possibly due to differing patient characteristics, study designs, or outcome ascertainment methods across smaller or higher-threshold studies. In contrast, age-based subgroups showed consistent associations and similar heterogeneity levels, indicating that age may not be a major source of heterogeneity.

The association between an elevated SIRI and poor prognosis in myocardial infarction is underpinned by a complex inflammatory immunopathological process. A high SIRI, indicative of elevated neutrophil and monocyte counts, directly contributes to atherosclerotic plaque instability. Plaque rupture is facilitated through several key mechanisms: the release of neutrophil extracellular traps (NETs) (30, 31), the degradation of the fibrous cap by matrix metalloproteinases (MMPs, such as MMP-9), and the generation of reactive oxygen species (ROS) (32–34). Simultaneously, monocytes infiltrate the vascular wall, differentiate into macrophages, and phagocytose lipids to form foam cells, exacerbating local inflammation through the secretion of pro-inflammatory factors like TNF-α and IL-1β (35). Furthermore, revascularization (e.g., PCI) following MI induces reperfusion injury, and a high SIRI signifies a heightened inflammatory response that may aggravate this damage. These inflammatory cells also interact to promote a pro-thrombotic state, thereby increasing the risk of stent thrombosis and recurrent MI.

In summary, a high SIRI reflects a systemic inflammatory state dominated by neutrophil and monocyte activation, coupled with impaired lymphocyte-mediated immunoregulation. This state contributes to the poor prognosis of myocardial infarction through multiple parallel pathways, including the destruction of plaque stability, aggravation of reperfusion injury, interference with cardiac reparative remodeling, and the promotion of thrombosis. Although SIRI is not traditionally recognized as a diagnostic criterion for AMI, its strong association with the condition enables it to complement established risk stratification tools such as the Killip classification and the GRACE score. By integrating SIRI into clinical assessment, a more comprehensive evaluation of patient risk can be achieved, thereby moderately enhancing the predictive accuracy for mortality and major adverse cardiovascular events. Moreover, for patients with less obvious symptoms in clinical practice (such as Killip class I or intermediate-risk in GRACE score), SIRI demonstrates added value in identifying individuals at higher risk, facilitating earlier and more intensive therapeutic interventions.

## 5 Limitations

While this meta-analysis provides comprehensive evidence supporting the prognostic value of SIRI in patients with myocardial infarction, several important limitations should be acknowledged and considered when interpreting the results.

First, the geographic and ethnic representation of the included studies is narrow. The vast majority were conducted in Chinese populations, with only three comparative groups from other regions (the USA and Italy). This predominance limits the generalizability of our findings to other ethnicities and healthcare settings, where genetic backgrounds, inflammatory profiles, and clinical management may differ substantially. Second, we observed substantial statistical heterogeneity in several meta-analyses, particularly for MACE (*I*^2^ = 90%) and the Gensini score (*I*^2^ = 97%). Although subgroup analyses were performed—suggesting that sample size and SIRI cut-off values may partly explain the variability—significant residual heterogeneity persists. Therefore, future studies based on more consistent SIRI thresholds and prospective designs would help reduce such heterogeneity, thereby providing more precise and generalizable evidence for risk assessment. Third, the definitions of the primary outcome, MACE, varied meaningfully across studies in terms of included components (e.g., whether heart failure, revascularization, or arrhythmias were consistently incorporated). This variability introduces potential outcome misclassification and heterogeneity, which could not be fully explored through subgroup analysis due to insufficient reporting in the original articles. We have therefore emphasized this as an important source of clinical and methodological heterogeneity. Fourth, the SIRI cut-off values used to define “high” vs. “low” SIRI groups differed widely across studies (ranging from 0.13 to 7.8), and no standardized or consensus-based threshold currently exists. Although we respected each study's original cut-off in our analysis, this variability complicates clinical interpretation and translation, representing a major barrier to the implementation of SIRI in routine practice. Fifth, all included studies were observational in design. While they consistently report an association between elevated SIRI and worse outcomes, residual confounding cannot be excluded, and causality cannot be inferred. Furthermore, for the MACE outcome, Egger's test indicated potential publication bias (*p* = 0.0001), suggesting that smaller studies with null results might be underrepresented. Although sensitivity analyses supported the robustness of the findings, the possibility of bias warrants a more cautious interpretation of the strength of association.

Finally, while SIRI emerges as a promising prognostic marker, current evidence does not yet support its use as a validated tool for guiding clinical decisions. Future studies—especially prospective, multi-ethnic cohorts with standardized SIRI thresholds and outcome definitions—are needed to confirm these findings and assess the clinical utility of SIRI in risk stratification and tailoring of management.

## 6 Conclusion

In conclusion, this systematic review and meta-analysis demonstrates that an elevated Systemic Inflammation Response Index (SIRI) is moderately and consistently associated with an increased risk of adverse clinical outcomes in patients with myocardial infarction, including major adverse cardiovascular events, all-cause mortality, stroke, subsequent AMI, and greater coronary artery disease severity. These findings underscore the robust prognostic value of SIRI, a readily accessible and cost-effective inflammatory biomarker derived from routine blood counts. The findings indicate that SIRI may be a useful tool for risk stratification, helping to identify MI patients at higher risk who could benefit from closer monitoring and individualized management strategies. Future studies should explore establishing standardized SIRI cut-off values and investigating whether SIRI can guide targeted anti-inflammatory therapies to ultimately improve long-term cardiovascular prognosis.

## Data Availability

The original contributions presented in the study are included in the article/Supplementary Material, further inquiries can be directed to the corresponding author/s.
