# Supplementary material for: Association between the systemic inflammation response index and the prognosis of patients with myocardial infarction: a systematic review and meta-analysis
Source: Front Cardiovasc Med. 2026 Feb 16;13:1755442. doi: 10.3389/fcvm.2026.1755442 (PMC12950576; doi:10.3389/fcvm.2026.1755442)
Supplement: Supplementary file 1 [file Table1.docx]

**SUPPLEMENTARY MATERIALS**

**Supplementary Table 1 Search strategy**

Entry terms

Myocardial Infarctions; Heart Attack; Heart Attacks; Myocardial Infarct; Myocardial Infarcts; Cardiovascular Stroke; Cardiovascular Strokes

Pubmed-151

(("Myocardial Infarction"[Mesh]) OR (((((((Myocardial Infarctions) OR (Heart Attack)) OR (Heart Attacks)) OR (Myocardial Infarct)) OR (Myocardial Infarcts)) OR (Cardiovascular Stroke)) OR (Cardiovascular Strokes))) AND ((systemic inflammation response index) OR (SIRI))

Embase-109

((Myocardial Infarction or (Myocardial Infarctions or Heart Attack or Heart Attacks or Myocardial Infarct or Myocardial Infarcts or Cardiovascular Stroke or Cardiovascular Strokes)) and (systemic inflammation response index or SIRI)).af.

Cochrane-2

((Myocardial Infarction or (Myocardial Infarctions or Heart Attack or Heart Attacks or Myocardial Infarct or Myocardial Infarcts or Cardiovascular Stroke or Cardiovascular Strokes)) and (systemic inflammation response index or SIRI)).af.

Web of science-170

((Myocardial Infarction) OR (((((((Myocardial Infarctions) OR (Heart Attack)) OR (Heart Attacks)) OR (Myocardial Infarct)) OR (Myocardial Infarcts)) OR (Cardiovascular Stroke)) OR (Cardiovascular Strokes))) AND ((systemic inflammation response index) OR (SIRI)) (Topic)

**Supplementary Table 2 NOS scale**

| Supplementary Table 2. Quality evaluation of the eligible studies with Newcastle–Ottawa scale. | | | | | | | | | |
| --- | --- | --- | --- | --- | --- | --- | --- | --- | --- |
| Cohort Study | Selection | | | | Comparability | | Outcome | | |
|  | Representative-ness | Selection of  non-exposed | Ascertainment  of exposure | Outcome not present at start | Comparability on most important factors | Comparability on other risk factors | Assessment of outcome | Long enough follow-up (median≥1 year) | Adequacy  (completeness) of follow-up |
| Jin Z 2021 | * | * | * | * | * | * | * | * | - |
| Han K 2022 | * | * | * | * | * | * | * | * | - |
| Li Y 2023 | * | * | * | * | * | - | * | * | * |
| Liu Y 2023 | * | * | * | * | * | - | * | * | - |
| Qu C 2023 | * | * | * | * | * | - | * | * | * |
| Wang Y 2023 | * | * | * | * | * | * | * | * | * |
| Wei X 2023 | * | - | * | * | * | * | * | - | - |
| Guo J 2024 | * | * | * | * | * | - | * | * | * |
| Hou H 2024 | * | * | * | * | * | - | * | * | - |
| Ma Y 2024 | * | * | * | * | * | * | * | * | - |
| Marchi F 2024 | * | * | * | * | * | * | * | * | * |
| Zhou H 2024 | * | * | * | * | * | - | * | * | * |
| He Q 2025 | * | * | * | * | * | * | * | * | * |
| *indicates criterion met; - indicates significant of criterion not met. | | | | | | | | | |

**Supplementary Table 3 Subgroups analysis**

| Subgroup | MACE | | | |
| --- | --- | --- | --- | --- |
|  | Comparative groups | OR [95%CI] | P value | I2 |
| **Total** | 12 | 1.42 [1.27, 1.58] | ＜0.0001 | 90% |
| Mean/median age |  |  |  |  |
| > 60y | 5 | 1.40 [1.19, 1.64] | ＜0.0001 | 94% |
| ≤ 60y | 7 | 1.50 [1.26, 1.79] | ＜0.0001 | 78% |
| Sample size |  |  |  |  |
| ≥ 960 | 7 | 1.25 [1.16, 1.35] | ＜0.0001 | 57% |
| < 960 | 5 | 1.86 [1.36, 2.55] | ＜0.0001 | 95% |
| SIRI cut-off |  |  |  |  |
| > 2 | 5 | 1.85 [1.35, 2.53] | ＜0.0001 | 94% |
| ≤ 2 | 6 | 1.40 [1.20, 1.64] | ＜0.0001 | 75% |
